# *N*-acetylgalactosaminyl transferase-3 is a potential new marker for non-small cell lung cancers

**DOI:** 10.1038/sj.bjc.6600536

**Published:** 2002-09-23

**Authors:** H Dosaka-Akita, I Kinoshita, K Yamazaki, H Izumi, T Itoh, H Katoh, M Nishimura, K Matsuo, Y Yamada, K Kohno

**Affiliations:** Department of Medical Oncology, Hokkaido University Graduate School of Medicine, North 15, West 7, Kita-ku, Sapporo, 060-8638, Japan; First Department of Medicine, Hokkaido University School of Medicine, Sapporo, 060-8638, Japan; Department of Molecular Biology, University of Occupational and Environmental Health, Japan, School of Medicine, Kitakyushu, 807-8555, Japan; Department of Surgical Pathology, Hokkaido University Medical Hospital, Sapporo, 060-8648, Japan; Department of Surgical Oncology, Hokkaido University Graduate School of Medicine, Sapporo, 060-8638, Japan; Hanno Research Center, Taiho Pharmaceutical Co. Ltd., Saitama, 357-0041, Japan

**Keywords:** GalNAc-T3, lung cancer, adenocarcinoma, Ki-67, prognosis

## Abstract

*N*-acetylgalactosaminyl transferase-3 (GalNAc-T3) is an enzyme involved in the initial glycosylation of mucin-type *O*-linked proteins. In the present study, we used immunohistochemistry to examine GalNAc-T3 expression in 215 surgically resected non-small cell lung cancers. We analysed the biological and clinical importance of GalNAc-T3 expression, especially with regard to its potential as a prognostic factor. We found that normal bronchial epithelial cells, bronchial gland cells, and alveolar pneumocytes showed cytoplasmic immunostaining for GalNAc-T3. Low expression of GalNAc-T3, observed in 93 of 215 tumours (43.4%), was found more frequently in tumours from smokers than those from nonsmokers (*P*=0.001), in squamous cell carcinomas than nonsquamous cell carcinomas (*P*<0.0001), and in moderately and poorly differentiated tumours than well differentiated tumours (*P*=0.0002). Multivariate logistic regression analysis showed that an association of low GalNAc-T3 expression with squamous cell carcinomas was the only one significant relationship of GalNAc-T3 expression with various factors (*P*<0.0001). Moreover, tumours losing GalNAc-T3 expression had a significantly higher Ki-67 labelling index than tumours retaining GalNAc-T3 expression (*P*=0.0003). Patients with low GalNAc-T3 expression survived a significantly shorter time than patients with high GalNAc-T3 expression in 103 pStage I non-small cell lung cancers (5-year survival rates, 58% and 78%, respectively; *P*=0.02 by log-rank test) as well as in 61 pStage I nonsquamous cell carcinomas (5-year survival rates, 63% and 85%, respectively; *P*=0.03). Low GalNAc-T3 expression was an unfavourable prognostic factor in pStage I non-small cell lung cancers (hazards ratio, 2.04; *P*=0.03), and in pStage I nonsquamous cell carcinomas (hazards ratio, 2.70; *P*=0.03). These results suggest that GalNAc-T3 is a new marker of non-small cell lung cancers with specificity for histology and prognosis.

*British Journal of Cancer* (2002) **87**, 751–755. doi:10.1038/sj.bjc.6600536 www.bjcancer.com

© 2002 Cancer Research UK

## 

Lung cancer is one of the leading causes of cancer death worldwide. Although the management and treatment of non-small cell lung cancers (NSCLCs) have improved, there is no evidence that therapeutic advances have resulted in a marked increase of survival rates. The overall 5-year survival rate remains at less than 15% (Ginsberg *et al*, 2001). It is not fully understood why patients with comparable stages of NSCLC may have different clinical courses and respond differently to similar treatments. A more sophisticated understanding of the pathogenesis and biology of these tumours (Hanahan and Weinberg, 2000; Sekido *et al*, 2001) could provide useful information for predicting clinical outcome, individualising treatment (Harpole *et al*, 1995; Strauss *et al*, 1995; Kwiatkowski *et al*, 1998; Dosaka-Akita *et al*, 2001), and identifying molecular targets of the treatment (Gibbs, 2000).

Oligosaccharides on glycoproteins are altered in tumorigenesis. These oligosaccharides often play a role in the regulation of the biological characteristics of tumours in terms of invasion and metastatic potential (Hakomori, 1989). Each oligosaccharide is synthesised by a specific glycosyltransferase (Varki, 1993). The initial glycosylation of mucin-type *O*-linked proteins is catalysed by one of the UDP-*N*-acetyl-α-D-Galactosamine: polypeptide *N*-acetylgalactosaminyl transferases (GalNAc-transferase family of enzymes) (Hagen *et al*, 1993; Homa *et al*, 1993; Wandall *et al*, 1997;). Three distinct human GalNAc-transferases, GalNAc-T1, GalNAc-T2, and GalNAc-T3, have been characterised (White *et al*, 1995; Bennett *et al*, 1996; Wandall *et al*, 1997). Recently another 3 homologue enzymes, GalNAc-T4, GalNAc-T5, and GalNAc-T6, have been identified (Bennett *et al*, 1996, 1998, 1999; Ten Hagen *et al*, 1998). Compared with the expression of GalNAc-T1 and GalNAc-T2, the expression of GalNAc-T3 is highly tissue specific. GalNAc-T3 mRNA has been detected in organs that contain secretory epithelial glands (Hagen *et al*, 1993; Homa *et al*, 1993; White *et al*, 1995; Bennett *et al*, 1996). It is hypothesised that the differential expression of GalNAc-T3 may affect the specialised functions of glycoproteins produced by normal and malignant cells, and that it is also associated with biological properties of normal and malignant cells (Sutherlin *et al*, 1997). However, GalNAc-T3 expression has not been previously examined in human lung cancers, or in human bronchial epithelial cells and alveolar pneumocytes, from which lung cancers develop.

In the present study, GalNAc-T3 expression was examined by immunohistochemistry in surgically resected NSCLCs. We analysed the biological and clinical importance of GalNAc-T3 expression, especially with regard to its potential as a prognostic factor.

## MATERIALS AND METHODS

### Tumour specimens and survival data

Primary tumour specimens from 215 NSCLCs were consecutively obtained by surgery from the Hokkaido University Medical Hospital during 1976 and 1994. The patients with NSCLCs consisted of 142 men and 73 women. The histologic classification of the tumour specimens was based on World Health Organization criteria (World Health Organization, 1982). The tumour specimens included 87 squamous cell carcinomas, 110 adenocarcinomas, nine large cell carcinomas, and eight adenosquamous cell carcinomas. Nonsquamous cell carcinoma included adenocarcinoma, large cell carcinoma and adenosquamous cell carcinoma. There were 119 Stage I, 18 Stage II, 70 Stage IIIa, one Stage IIIb, and seven Stage IV tumours. The postsurgical pathologic TNM stage (pTNM) was determined according to the guidelines of the American Joint Committee on Cancer (American Joint Committee on Cancer, 1992). Of the 119 patients with pStage I tumours resected with curative intent, survival was analysed for the 103 patients who met the following criteria: (1) survived for more than 3 months after surgery; (2) did not die of causes other than lung cancer within 5 years after surgery; and (3) were followed for more than 3 years after surgery (for patients who remained alive). Sixteen patients did not meet the above criteria (four died within 3 months after surgery, five died of causes other than lung cancer within 5 years, and seven had no survival records after surgery) were excluded from the survival analysis. Of the 103 patients for whom survival was analysed, 54 patients had died of cancer, including 27 with squamous cell carcinomas, 22 with adenocarcinomas, three with large cell carcinomas and two with adenosquamous cell carcinomas. The Karnofsky performance status was 90% or greater in all these 103 patients. This study was approved by the Medical Ethical Committees of Hokkaido University School of Medicine. Because all patients were coded, they could not be individually identified.

### Construction of the plasmid and preparation of GST fusion protein

The GalNAcT3 cDNA was cloned into pGEM-Teasy (Promega, Madison, WI, USA) by RT–PCR using the primers 5′-ATGGCTCACCTAAAGCGACTAG-3′ and 5′-GAAAGACTCCAGTCAAAATTTCC-3′, as described previously (Nomoto *et al*, 1999). The *Eco*RI-*Eco*47III fragment (amino acid residues 1–178 of the whole 324 amino acids) was cloned into *Eco*RI-*Sma*I sites in the pGEX-6P, and GST fusion protein (GST-GalNAcT3N) was purified according to the manufacturer's protocol(Pharmacia, Uppsala, Sweden).

### Western blot analysis of GST fusion protein

Both GST and GST-GalNAcT3N were separated on a 12% SDS–PAGE gel and transferred to polyvinylidene difluoride membrane (Millipore, Bedford, MA, USA) using semidry blotter. Immunoblot analysis was performed with anti-GST antibody (Santa Cruz Biotechnology, Santa Cruz, CA, USA) and anti-GalNAcT3 antibody, which was a rabbit polyclonal antibody against a synthesised peptide of human GalNAc-T3 (Nomoto *et al*, 1999) and was used for immunohistochemistry.

### Immunohistochemistry for GalNAc-T3 expression

GalNAc-T3 expression was analysed by immunohistochemistry. The labelled streptavidin biotin method was used on 4-μm sections of formalin-fixed, paraffin-embedded tissues after deparaffinisation. Briefly, deparaffinised tissue sections were microwaved twice in 10 mM citrate buffer (pH 6.0) for 5 min to retrieve the antigenicity. The sections were incubated with normal rabbit serum to block the non-specific antibody binding sites. The sections were then incubated with a 1 : 5000 dilution of rabbit polyclonal antibody against a synthesised peptide of human GalNAc-T3 (Nomoto *et al*, 1999) or with control rabbit non-immunised serum at 4°C overnight. Immunostaining was performed by the biotin-streptavidin immunoperoxidase method with 3,3′-diaminobenzidine as a chromogen (SAB-PO kit; Nichirei, Tokyo, Japan). Sections were counterstained with methyl green. GalNAc-T3 expression in normal bronchial epithelial cells, bronchial gland cells, and alveolar pneumocytes in the same sections served as an internal positive control. The GalNAc-T3 expression in tumours was classified as high or low, according to the proportion of positively stained tumour cells. Tumours with staining in at least 50% of cancer cells, were judged as having high GalNAc-T3 expression (retaining expression of GalNAc-T3). Tumours with staining in less than 50% of cancer cells, were judged as having low GalNAc-T3 expression (losing expression of GalNAc-T3), as we previously described (Shibao *et al*, 2002).

For the Ki-67 staining, the results that were previously reported (Hommura *et al*, 2000) were used for the present study.

### Statistical analysis

The associations between GalNAc-T3 expression and categorical variables were analysed by the χ^2^ test or Fisher's exact test as appropriate. The associations between GalNAc-T3 expression and age or the Ki-67 labelling index (LI) were analysed by Student's *t*-test. To simultaneously examine the effect of more than one factor on GalNAc-T3 expression, multivariate logistic regression analysis was used (Cox and Snell, 1989). The survival curves were estimated using the Kaplan-Meier method, and differences in survival distributions were evaluated by the generalised Wilcoxon test. Cox's proportional hazards modelling of factors potentially related to survival was performed to identify factors with a significant influence on survival. *P* values less than 0.05 were considered statistically significant. All tests were two-sided.

## RESULTS

**Figure 1:**
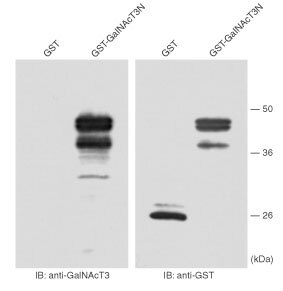
Western blotting of GST fusion protein. GST (100 ng) and GST-GalNAcT3N (200 ng) proteins were loaded on a 12% SDS–PAGE gel and transferred to membrane. Immunoblot analysis was performed with an anti-GalNAcT3 antibody (left) and anti-GST antibody (right). IB, immunoblotting.

**Figure 2:**
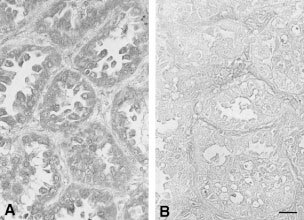
Immunoreactivity of an anti-GalNAc-T3 polyclonal antibody. Immunostaining was performed with this antibody in a NSCLC specimen after incubation of this antibody with excess of GST (**A**) or GST-GalNAcT3N (**B**). Scale bar=20 μm.

To confirm the availability of an anti-GalNAc-T3 polyclonal antibody, Western blot analysis of a GST fusion protein (GST-GalNAcT3N) was performed (Figure 1

). Strong signals of the GST fusion protein were obtained in the immunoblotting with anti-GalNAc-T3 polyclonal antibody as well as in that with anti-GST antibody. The specificity of this anti-GalNAc-T3 polyclonal antibody was also tested by immunohistochemistry. After incubation of this antibody with the excess of GST-GalNAcT3N, the positive immunostaining was abolished (Figure 2

).

**Figure 3:**
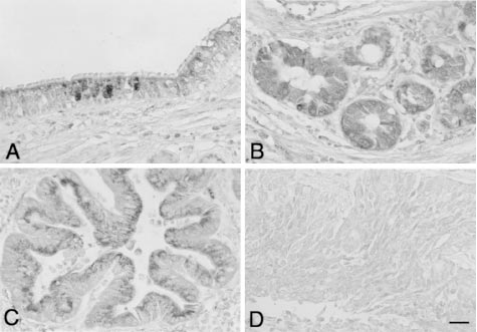
Immunohistochemical staining patterns for GalNAc-T3. Normal bronchial epithelial cells (**A**) and bronchial gland cells (**B**) showed GalNAc-T3 expression. GalNAc-T3 expression was found diffusely in the cytoplasm of tumour cells or localised in the Golgi apparatus in an adenocarcinoma (**C**), but not seen in a squamous cell carcinoma (**D**). Scale bar=20 μm.

Typical immunostaining patterns for GalNAc-T3 with this antibody in NSCLCs are shown in Figure 3

. In tumour cells, GalNAc-T3 expression was found diffusely in the cytoplasm, or localised in the Golgi apparatus. Normal bronchial epithelial cells (Figure 3A), bronchial gland cells (Figure 3B), and alveolar pneumocytes (data not shown) also expressed GalNAc-T3.

Of the 215 NSCLCs, 122 (56.7%) had high GalNAc-T3 expression, and 93 (43.3%) had low GalNAc-T3 expression (Table 1

**Table 1:**
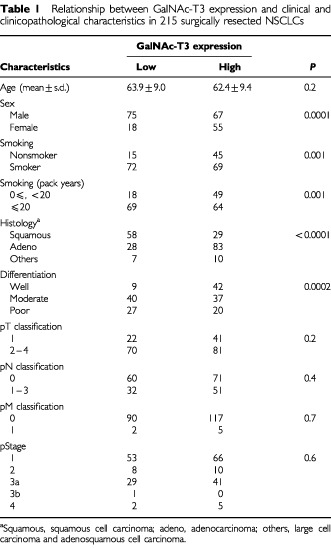
Relationship between GalNAc-T3 expression and clinical and clinicopathological characteristics in 215 surgically resected NSCLCs

). The status of GalNAc-T3 expression was statistically analysed to investigate possible correlations with clinical and clinicopathological characteristics of NSCLCs. Low expression of GalNAc-T3 was significantly more prevalent in tumours from men than in those from women (*P*=0.0001 by the χ^2^ test) and in tumours from smokers compared to nonsmokers (*P*=0.001). Low GalNAc-T3 expression was also more prevalent in squamous cell carcinomas than nonsquamous cell carcinomas (*P*<0.0001) (Table 1). GalNAc-T3 expression was not associated with pTNM classifications and pStage. Multivariate logistic regression analysis showed a significant association of low GalNAc-T3 expression with squamous cell (*P*<0.0001) (Table 2

**Table 2:**
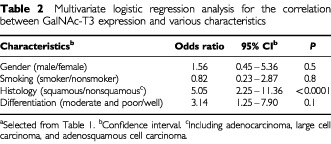
Multivariate logistic regression analysis for the correlation between GalNAc-T3 expression and various characteristics

). This relationship between GalNAc-T3 expression and histology was the only significant relationship between GalNAc-T3 expression and various factors within the context of the multivariate model. Tumours having low GalNAc-T3 expression showed a significantly higher Ki-67 LI than tumours having high GalNAc-T3 expression (*P*=0.0003) (Table 3

**Table 3:**
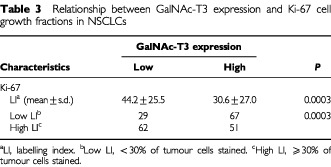
Relationship between GalNAc-T3 expression and Ki-67 cell growth fractions in NSCLCs

). Tumours having low GalNAc-T3 expression also exhibited a high Ki-67 LI more frequently than tumours having high GalNAc-T3 expression (*P*=0.0003).

**Figure 4:**
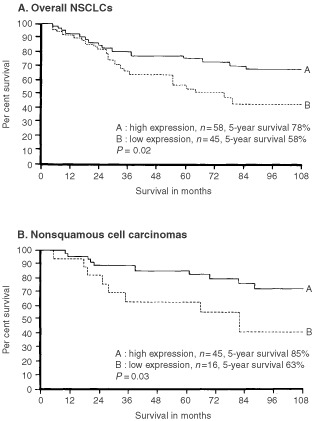
Kaplan–Meier survival curves of patients with pStage I NSCLCs stratified by GalNAc-T3 expression. Survival curves of patients with overall NSCLCs (**A**) and with nonsquamous cell carcinomas (**B**) are stratified by low and high GalNAc-T3 expression. Nonsquamous cell carcinoma included adenocarcinoma, large cell carcinoma and adenosquamous cell carcinoma.

We analysed the relationship between GalNAc-T3 expression and postsurgical survival in pStage I NSCLCs (Figure 4

). When the 103 pStage I NSCLCs were evaluated together, patients with tumours having low GalNAc-T3 expression survived a significantly shorter time than patients with tumours having high expression (5-year survival rates, 58% and 78%, respectively, by the Kaplan-Meier method; *P*=0.02 by the generalised Wilcoxon test) (Figure 4A). When only patients with 43 squamous cell carcinomas were analysed, GalNAc-T3 expression did not show a significant effect on survival (5-year survival rates, 54% for low GalNAc-T3 and 57% for high GalNAc-T3; *P*=0.8). Among 61 nonsquamous cell carcinoma patients, patients with tumours having low GalNAc-T3 expression survived a significantly shorter time than patients with tumours having high expression (5-year survival rates, 63% and 85%, respectively; *P*=0.03) (Figure 4B).

The importance of GalNAc-T3 as a prognostic factor was analysed by the Cox's proportional hazards model analysis in patients with pStage I NSCLCs (Table 4

**Table 4:**
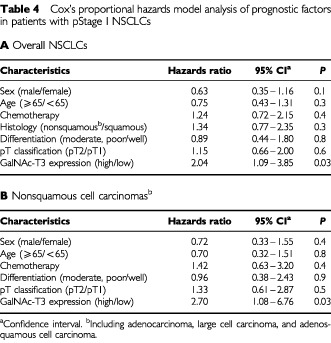
Cox's proportional hazards model analysis of prognostic factors in patients with pStage I NSCLCs**A** Overall NSCLCs

). Univariate analysis of potential prognostic factors revealed that low expression of GalNAc-T3 was the only statistically significant unfavourable prognostic factor in overall NSCLCs (hazards ratio, 2.04; *P*=0.03) and in nonsquamous cell carcinomas (hazards ratio, 2.70; *P*=0.03). GalNAC-T3 expression was not a prognostic factor in squamous cell carcinomas (hazards ratio, 0.91; *P*=0.8).

## DISCUSSION

In the present study, GalNAc-T3 expression was frequently decreased in NSCLCs, although it was expressed in normal bronchial epithelial cells, bronchial gland cells and alveolar pneumocytes. Furthermore, low GalNAc-T3 expression of NSCLCs was associated with a shorter survival period, and was an unfavourable prognostic factor.

The finding that GalNAc-T3 expression was retained more frequently in nonsquamous cell carcinomas (most of which were adenocarcinomas) than in squamous cell carcinomas may reflect tissue specific expression of GalNAc-T3 in organs that contain secretory epithelial glands (Hagen *et al*, 1993; Homa *et al*, 1993; White *et al*, 1995; Bennett *et al*, 1996). Moreover, in nonsquamous cell carcinomas, decreased expression of GalNAc-T3 was associated with unfavourable prognosis. Consistent with these results, we have recently found that GalNAc-T3 is expressed in normal epithelial cells and gland cells of the colon, and that decreased expression of GalNAc-T3 is an unfavourable prognostic factor in adenocarcinomas of the colon (Shibao *et al*, 2002).

The mechanism underlying the relationship between decreased GalNAc-T3 expression and poor prognosis remains to be determined. Bronchial and alveolar epithelia, from which NSCLCs develop, normally express GalNAc-T3. Decreased GalNAc-T3 expression may induce decreased level of glycosylation in certain types of glycoprotein, resulting in altered functions of the glycoproteins. Therefore, the biological importance of GalNAc-T3 expression depends on the function of target substrate glycoproteins. The importance of GalNAc-T3 expression for the development and progression of cancer as well as for maintaining physiologic properties of normal cells also remains to be determined, although certain cancer-associated decreases or increases in glycosylation has been shown to directly contribute to cellular transformation (Vavasseur *et al*, 1994; Demetriou *et al*, 1995). Collectively, decreased expression of GalNAc-T3 may directly contribute to altered biological properties of NSCLCs. In fact, in this study, low expression of GalNAc-T3 in NSCLCs was associated with a higher LI of Ki-67 cell growth fractions, and resulted in shorter survival times.

In conclusion, these results indicate that GalNAc-T3 is a new marker of NSCLCs with specificity for histology and survival. GalNAc-T3 expression may be useful to stratify patients with pStage I tumours into groups at high and low risks of recurrence in NSCLCs, especially in nonsquamous cell carcinomas.
